# Increased expression of glutamine transporter SNAT2/SLC38A2 promotes glutamine dependence and oxidative stress resistance, and is associated with worse prognosis in triple-negative breast cancer

**DOI:** 10.1038/s41416-020-01113-y

**Published:** 2020-10-08

**Authors:** Matteo Morotti, Christos E. Zois, Rokaya El-Ansari, Madeleine L. Craze, Emad A. Rakha, Shih-Jung Fan, Alessandro Valli, Syed Haider, Deborah C. I. Goberdhan, Andrew R. Green, Adrian L. Harris

**Affiliations:** 1 Hypoxia and Angiogenesis Group, Weatherall Institute of Molecular Medicine, Department of Oncology, University of Oxford, Oxford, OX3 9DS UK; 2 Nottingham Breast Cancer Research Centre, Division of Cancer and Stem Cells, School of Medicine, University of Nottingham Biodiscovery Institute, Nottingham, NG7 2RD UK; 3 Department of Physiology, Anatomy and Genetics, Le Gros Clark Building, University of Oxford, South Parks Road, Oxford, OX1 3QX UK; 4 The Breast Cancer Now Toby Robins Research Centre, Division of Breast Cancer Research, The Institute of Cancer Research, London, UK

**Keywords:** Breast cancer, Cancer metabolism

## Abstract

**Background:**

Glutamine (Gln) is an abundant nutrient used by cancer cells. Breast cancers cells and particularly triple-receptor negative breast cancer (TNBC) are reported to be dependent on Gln to produce the energy required for survival and proliferation. Despite intense research on the role of the intracellular Gln pathway, few reports have focussed on Gln transporters in breast cancer and TNBC.

**Methods:**

The role and localisation of the Gln transporter SLC38A2/SNAT2 in response to Gln deprivation or pharmacological stresses was examined in a panel of breast cancer cell lines. Subsequently, the effect of SLC38A2 knockdown in Gln-sensitive cell lines was analysed. The prognostic value of SLC38A2 in a cohort of breast cancer was determined by immunohistochemistry.

**Results:**

SLC38A2 was identified as a strongly expressed amino acid transporter in six breast cancer cell lines. We confirmed an autophagic route of degradation for SLC38A2. *SLC38A2* knockdown decreased Gln consumption, inhibited cell growth, induced autophagy and led to ROS production in a subgroup of Gln-sensitive cell lines. High expression of SLC38A2 protein was associated with poor breast cancer specific survival in a large cohort of patients (*p* = 0.004), particularly in TNBC (*p* = 0.02).

**Conclusions:**

These results position SLC38A2 as a selective target for inhibiting growth of Gln-dependent breast cancer cell lines.

## Background

The significance of amino acid (AA) metabolism and glutamine (Gln, the most abundant AA in blood serum) in tumour progression has been well recognised, leading to intense interest in therapeutic applications.^1^ The maintenance of high levels of AA in cancer cells provides a ready source of carbon and nitrogen to support biosynthesis, energetics and cellular homoeostasis. AA levels are critical for regulating the balance between the positive anabolic actions of the mammalian target of rapamycin complex 1 (mTORC1) microenvironmental sensor and the effects of inhibiting mTORC1, leading to autophagy and catabolism.^2^

Breast cancer is a heterogeneous disease characterised by different morphology, clinical outcome, and response to treatment. From a metabolic point of view, Gln-dependent mechanisms also can vary substantially among breast cancer subtypes.^3^ Triple-negative breast cancer (TNBC), an aggressive form of breast cancer, is dependent on exogenous Gln for survival and growth^4,5^ and inhibitors of Gln transport and metabolism have been proposed as potential anti-tumour therapies.^6,7^

Gln is transported into cells through many amino acid transporters (AATs).^8^ A recently proposed classification by Broer et al., suggested that AATs can be divided into loaders, such as SLC38A1/SNAT1, mediating the net uptake of specific AAs;^9^ AAT harmonisers, such as SLC1A5/ASCT2 and SLC7A5/LAT1, which rapidly exchange different AAs and ensure that all AAs are present in the cytosol; and rescue AATs, such as SLC38A2/SNAT2 and SLC1A4/ASCT1 that provide redundant capacity and are upregulated under stress conditions.^10,11^ The role of Gln metabolism and transporters, including SLC38A2, in mediating resistance to anti-endocrine therapies has been recently demonstrated.^12–14^ However, despite the high-dependency on Gln for TNBC growth, the role of Gln transporters in this subtype has been less investigated.^15^ As there are many Gln transporters, we investigated which were most abundant and their relationship to Gln dependence.

We found the Gln transporter SLC38A2/SNAT2 to have the highest expression level in many breast cancer cell lines. Consequently, we investigated and subsequently demonstrated its role in adaptation to Gln starvation in Gln-sensitive cell lines, including TNBC cells. High SLC38A2 levels were associated with poor clinical outcome in TNBC patients, suggesting a key metabolic role for this transporter.

## Methods

### Cell culture

Cell lines were obtained from ATCC (MCF7, T47D, SKBR3, HCC1806, MDA-MB-231, MDA-MB-468), and they were authenticated six months prior to the first submission of the manuscript. All cell lines were maintained in DMEM 25 mM glucose supplemented with 10% FBS and no antibiotics. All the experiments were performed in 5 mM glucose and 10% dialysed FBS was used. For spheroid culture, we plated 5000 cells into ultra-low–adherent round-bottom 96-well plates (VWR) with Matrigel, except for MDA-MB-231, which did not need Matrigel.

### Cell proliferation

Cells (1 × 10^5^ cells per well) were seeded in triplicate on six-well plates. *N*-acetylcysteine was added at 10 mM and replaced every 2 days. Trypsinised cells were counted using the Auto T4 Cell Counter (Nexcelom Bioscience). We used α-(Methylamino) isobutyric acid (MeAIB) (M2383, Sigma-Aldrich) at 10 mM concentration for 2D and spheroid culture. Treatment was started on day 3 and renewed every 2–3 days. Images were captured every 2–3 days.

### RNA extraction and qRT-PCR

Cells were lysed in Trizol reagent (Invitrogen) and RNA was extracted using ethanol precipitation. The RNA quality and quantity were confirmed using the NanoDrop ND-1000 spectrophotometer. Reverse transcription (RT) was performed using the High Capacity cDNA RT kit (Applied Biosystems). qRT-PCR was performed using the SensiFAST SYBR No-ROX Kit (Bioline). Raw data were analysed using *Ribosomal Protein L11 (RPL11)* and *β-actin* as housekeeping genes. Each PCR reaction was performed in triplicate. Primer sequences are reported in Table 1.

**Table 1:** Oligonucleotides used for RT-PCR.

Gene Name	Forward	Reverse
β-ACTIN	CCCAGCACAATGAAGATCAA	CGATCCACACGGAGTACTTG
RPL11	CTTTGGCATCCGGAGAAAT	TCCAAGATTTCTTCTGCC TTG
SLC1A4	TGTTTGCTCTGGTGTTAGGAGT	CGCCTCGTTGAGGGAATTGAA
SLC1A5	GAGCTGCTTATCCGCTTCTTC	GGGGCGTACCACATGATCC
SLC7A5	CCGTGAACTGCTACAGCGT	CTTCCCGATCTGGACGAAGC
SLC38A1	TGACAGTGCCCGAGGATGATA	AGACATGCCTAAGGAGGTTGTA
SLC38A2	ATGAGTTGCCTTTGGTGATCC	ACAGGACACGGAACCTGAAAT

### Gene silencing by RNA interference

Reverse transfection of siRNA duplexes (20 nM) was undertaken in Optimem using Lipofectamine RNAiMax (Invitrogen) according to the manufacturer’s instructions. The siRNA duplexes targeting SLC38A2 (ON-TARGETplus SMARTpool, LQ-007559–01–0002) or scrambled control were used.

### Immunofluorescence

Immunostaining was performed as previously reported.^12^ Samples were incubated overnight with SLC38A2 (G-8, SC166366), LAMP1 (CST9091), LAMP2 (CST49067), proteasome 20S LMP2 (ab3328), Rab5 (CST3547), TGN46 (AHP500G) primary antibodies and then incubated with fluorescently conjugated secondary antibodies or Alexa Fluor® 594 phalloidin (A12381) and DAPI for 1 h at 37 °C. The samples were imaged on a Zeiss LSM 680 or 780 confocal microscope.

### Determination of glutamine consumption

Cells (1 × 10^4^/well) in a 24-well plate were cultured for 5 days, the medium was collected, and cells were lysed with RIPA buffer (Sigma-Aldrich). Concentrations of Gln in the medium and in the cell lysate were determined with the Gln Detection Assay Kit (ab197011; Abcam) following manufacturer’s guidelines. Gln consumption was calculated as (Gln in normal medium - Gln in the medium after culturing cells) and normalised to the protein level.

#### Measurement of reactive oxygen species

Reactive oxygen species (ROS) after SLC38A2 knockdown in normal medium (4 mM) or low-Gln medium (1 mM) was measured after 5 days using the Cellular ROS Assay Kit (ab186027; Abcam). The procedure was performed following the manufacturer’s protocol and fluorescence was measured at 590 nm emission.

### Immunoblotting

Protein concentrations were quantified using a BCA protein assay kit (ThermoFisher Scientific). Samples containing 35 μg of protein in NuPAGE™ LDS Sample Buffer (4X) were separated using 8–12% pre-cast SDS-PAGE gels (BioRad). Proteins were transferred to PVDF membranes and blocked for 1 h in 5% TBST/milk. Primary antibodies were all used at 1:1000. Rabbit anti- SLC38A2 (BMP081), SLC7A5/LAT1 (CST5347), SLC1A5/ASCT2 (CST8057), SLC38A1/SNAT1 (CST36057), xCT/SLC7A11 (CST12691), LAMP1 (CST9091), LAMP2 (CST49067), AMPKα (CST5832), Phospho-Thr172-AMPKα (CST2535), Acetyl-CoA Carboxylase (ACC; CST3676), Phospho-Ser79-ACC (CST11818), phospho-Ser2448-mTOR (CST2971), Anti-SQSTM1/p62 (ab56416), ULK1 (CST8054), phospho-Ser555-ULK1 (CST5869). Appropriate secondary horseradish peroxidase–linked antibodies were used (Dako). Blots were developed on ImageQuant LAS4000 using ECL plus reagent (GE Healthcare).

### Tissue microarrays and immunohistochemical analysis

The set (*n* = 1685) was arrayed as previously described.^16^ Immunohistochemical (IHC) staining was performed on 4 µm tissue microarrays (TMA) sections using the Novolink polymer detection system (Leica Biosystems) as previously described.^17^ Briefly, tissue sections were incubated 1 h at room temperature with the primary SLC38A2 antibody (1:25). Slides were washed and incubated with post-primary block for 30 min. Novolink polymer was applied for 30 min: 3,3-diaminobenzidine chromogen was applied for 5 min. Slides were then counterstained with haematoxylin for 6 min.

Stained TMA sections were scored using high-resolution digital images (NanoZoomer; Hamamatsu Photonics), at ×20 magnification. Evaluation of staining for SLC38A2 was based on a semi-quantitative assessment of digital images of the cores using a modified histochemical score (H-score) which included assessment of both the intensity and the percentage of stained cells. Staining intensity was assessed as follows: 0, negative; 1, weak; 2, medium; 3, strong and the percentage of the positively stained tumour cells was estimated subjectively. The final H-score was calculated by multiplying the percentage of positive cells (0–100) by the intensity (0–3). Dichotomisation of protein expression in predicting breast cancer specific survival (BCSS) was determined using x-tile software (http://tissuearray.org).

IHC staining and dichotomisation of the other biomarkers were as per previous publications.^18^ ER and progesterone receptor (PgR) positivity was defined as ≥1% staining. Immunoreactivity of HER2 was scored using standard HercepTest guidelines (Dako) and by chromogenic in situ hybridisation in borderline cases. Breast cancer molecular subtypes were defined based on tumour IHC profile and the Elston-Ellis mitotic score as: ER ± HER2- low proliferation (mitotic score 1), ER ± HER2- high proliferation (mitotic score 2 and 3); HER2-positive class: HER2 + regardless of ER status; triple negative: ER−, PgR− and HER2−.

### Bioinformatics analysis

The Metabric breast cancer dataset was pre-processed, summarised and quantile-normalised from the raw expression files generated by Illumina BeadStudio (R packages: beadarray v2.4.2 and illuminaHuman v3.db_1.12.2). Raw files were downloaded from European genome-phenome archive (Study ID: EGAS00000000083). Correlation analysis and visualisations were performed in R statistical environment version 3.4.4.

### Statistical analysis

Statistical analysis and graphs were performed using GraphPad Prism v6.0. Results are plotted as mean values with standard deviation (SD). Statistical tests and the number of repeats are described in the figure legends. Student’s *t*-test was used for two sample analyses and normal distributions were assumed; otherwise, the non-parametric Mann–Whitney test was used. Analysis of variance was used for >2 sample analyses. The chi-square test was performed for categorical variables. No samples or experimental repeats were excluded from analyses.

Survival curves were analysed by the Kaplan–Meier and log-rank test. A *p*-value < 0.05 was considered significant. The study endpoints were 10-year BCSS.

## Results

### SLC38A2 mRNA is highly expressed in breast cancer cell lines

**Fig. 1:**
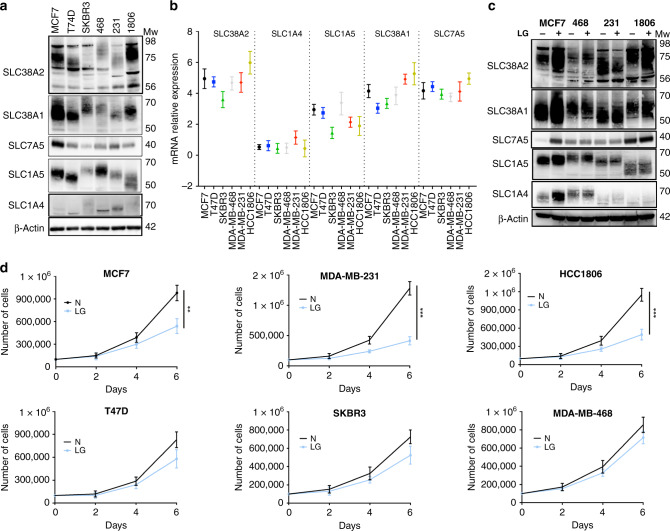
Glutamine dependency and glutamine transporters expression in breast cancer cell lines. **a** Immunoblotting of extracts from six different breast cancer cell lines to assess the levels of different glutamine (Gln) transporters. β-actin is shown as a loading control, *n* = 3. **b** Plot of *SLC38A2* mRNA levels (*Y*-axis) in six breast cancer cell lines (*X*-axis). Each dot represents the mean mRNA level of each *AAT* in a specific cell line after normalisation to housekeeping genes (*β-actin* and *RPL11*). *N* = 3. **c** Immunoblot analysis from MCF7, MDA-MB-468, MDA-MB-231 and HCC1806 cultured in normal (N, 4 mM) and in low glutamine medium (LG, 1 mM) for 24 h. **d** Breast cancer cell lines were grown in media with complete (N) or low Gln (1 mM, LG) in 10% dialysed FBS. The number of live cells per well was counted at day 2, 4 and 6. Error bars indicate SD. **P < 0.01, ***P < 0.001; unpaired *t*-test. *N* = 3.

We examined mRNA and protein levels of several transmembrane Gln transporters such as SLC7A5/LAT1, SLC1A4/ASCT1, SLC1A5/ASCT2, SLC38A1/SNAT1 and SLC38A2/SNAT2 in a variety of breast cancer cell lines representing the luminal (ER+, HER2−; MCF-7 and T47D), HER2+ (SKBR3), basal-like (HCC1806; MDA-MB-468, 468) and claudin-low (MDA-MB-231, 231) molecular subtypes of breast cancer (Fig. 1a). Different cell lines displayed heterogeneity in the mRNA and protein levels of several Gln transporters (Fig. 1a, b). Interestingly, *SLC38A2* mRNA was the most abundant transcript in many cell lines (Fig. 1b). Similarly, *SLC38A2* mRNA was also the most abundant transcript amongst a wide range of AAT in different breast cancer cell lines from the Cancer Cell Line Encyclopaedia (CCLE; Fig. S1A). Amongst the different cell lines, MCF7, MDA-MB-231 and HCC1806 had the highest levels of expression of several Gln transporters both at mRNA and protein levels (Fig. 1a, b).

We then cultured the same cell lines in a growth medium that contained high (4 mM) or lower levels of Gln (1 mM) and followed their growth for six days. The optimal growth of breast cancer cell lines MCF7, MDA-MB-231 and HCC1806 was dependent on Gln supplementation (Fig. 1c). In contrast, T47D, SKBR3, and MDA-MB-468 were significantly less dependent on Gln for growth (Fig. 1c). A smaller subgroup of four cell lines were also tested in 0.5 mM Gln levels (Fig. S1B). The results showed an increased sensitivity to lower Gln levels for MCF7, MDA-MB-231 and T47D but not SKBR3 (Fig. S1B) as previously shown.^4^

We then assessed whether the variable dependence of cancer cells on Gln could also be a consequence of altered expression of Gln transporter genes. A selected panel of cell lines having a higher sensitivity to Gln (MCF7, MDA-MB-231 and HCC1806) and the less-sensitive MDA-MB-468 were exposed to low Gln medium for 24 h, and the Gln transporter levels were assessed by qRT-PCR and WB. In two of the sensitive cell lines (MCF7 and HCC1806), the system A transporters (SLC38A1 and SLC38A2) increased after Gln deprivation both at mRNA and protein level, but levels remained unchanged in the less Gln-sensitive MDA-MB-468 and in the Gln-sensitive MDA-MB-231 cell line (Fig. 1c and Fig. S1C). Due to its high mRNA abundance, protein expression in Gln-sensitive cell lines and previous studies showing extensive transcriptional and post-translational regulation of SLC38A2 under microenvironmental stress conditions,^12,19^ we investigated in more detail the role of SLC38A2 under glutamine starvation and endoplasmic reticulum (ER) stress in breast cancer cell lines.

### SLC38A2 is re-localised from the trans–Golgi network under different stresses

SLC38A2 induction under extracellular AA deprivation, including Gln deprivation, has been previously linked to ATF4 upregulation (adaptive regulation).^20^ As different stresses are known to regulate protein localisation, and SLC38A2 has been demonstrated to undergo re-localisation from a cytosolic compartment towards the cell’s plasma membrane in anabolic conditions (such as insulin administration)^21^ and hyperosmotic stress,^22^ we investigated the localisation patterns of SLC38A2 in response to different stresses able to induce the ATF4 pathway, such as PP242 treatment (an ATP-competitive mTORC1/mTORC2 inhibitor),^23^ thapsigargin (TG) and energetic stress (24 h of AA starvation) in different breast cancer cell lines. Firstly, by two-colour super resolution-stimulated emission depletion (STED) microscopy we showed that SLC38A2 was located predominantly in the Trans–Golgi network (TGN) in MCF7 (Fig. S2A), consistent with previous analysis in normal and cancer cells.^22^ SLC38A2 localisation appeared typical of intracellular vesicles, present in proximity to the TGN and also other cytoplasmic regions (Fig. S2A). A specific antibody against the N-terminus of SLC38A2 was used for these experiments (Fig. S2B).

**Fig. 2:**
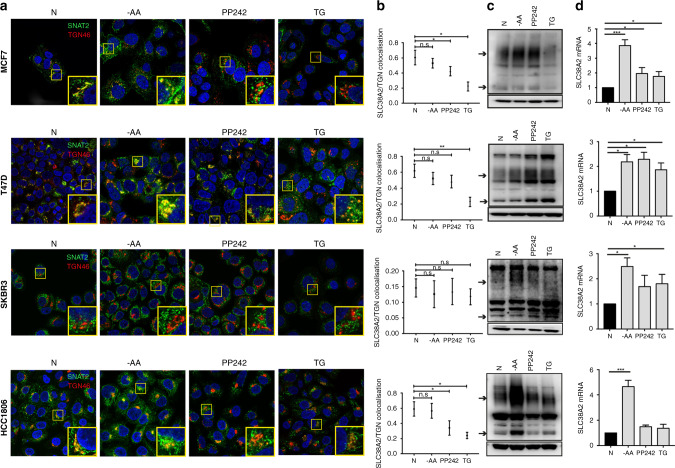
SLC38A2 undergoes re-localisation from the Trans–Golgi network under diverse stress in different breast cancer cell lines. Co-localisation study of SLC38A2 at TGN in different cell lines and after diverse treatments. **a** Representative confocal images of MCF7 (top row), T47D (second row), SKBR3 (third row), HCC1806 (fourth row). Cells were fixed and stained with SLC38A2 (green) and with TGN46 (red) in normoxia (left column) and after 24 h of: amino acid deprivation (EBSS medium, no AA, 10% dialysed FBS; second column), PP242 treatment (20 μm; third column) and thapsigargin treatment (8 h, fourth column, Scale bars 5 μm). **b** Pearson’s test co-localisation analysis of SLC38A2 at TGN46 during treatments. Scheme of the analysis procedure: Circular patches surrounding a cell or a group of cell or manual evaluation SLC38A2 levels (ROI) and non- SLC38A2 signal (random ROI) were selected. Co-localisation values were calculated using a pixel-wise Pearson’s test. Frequency quantification of Pearson’s test values (−1: opposing, 0: no and 1: maximum co-localisation) for SLC38A2 vs. TGN in MCF7 (top row), SLC38A2 vs. TGN in T47D (second row), SLC38A2 vs. TGN in SKBR3 (third row) and SLC38A2 vs. TGN in HCC1806 (fourth row). *N* = 3, one-way ANOVA, *p < 0.05, **p < 0.05. **c** Western blot analysis of protein expression in the same four cell lines after previously mentioned treatments. β-actin served as loading control. *N* = 3. Exposure time (HCC1806: 90 s; MCF7: 3 min; T47D: 5 min; SKBR3: 9 min. Arrows indicate predicted SLC38A2 protein) **d** The relative expression of *SLC38A2* total mRNA, in MCF7, T47D, SKBR3 and HCC1806 after different treatment as above, was analysed. Results were obtained by using the mean of the Ct values of *SLC38A2* transcript after normalisation to housekeeping genes *(β-actin* and *RPL11*). Error bars, SD; ANOVA *t*-test. *p < 0.05, ***p < 0.001; *N* = 3.

AA deprivation increased *SLC38A2* mRNA and protein levels, particularly in the Gln-sensitive cell lines (MCF7 and HCC1806) (Fig. 2a–d) with no re-localisation from the TGN (Fig. 2b). PP242 treatment increased *SLC38A2*mRNA and protein in the ER+ cell lines (MCF7, T47D) but not in SKBR3 and HCC1806 cell lines (Fig. 2c, d). Moreover, re-localisation from the TGN was seen in MCF7, T47D and HCC1806 cell lines (Fig. 2b). SKBR3 could not be assessed as SLC38A2 is not in the TGN of that cell line (Fig. 2a, b). Similar results were also confirmed in the MDA-MB-231 TNBC cell line (Fig. S2C). Interestingly, TG treatment induced an increase of *SLC38A2* mRNA in MCF7, T47D and SKBR3 (Fig. 2d), but a decrease in SLC38A2 protein in MCF7 and HCC1806, as previously seen,^24^ with re-localisation from the TGN (Fig. 2b, c). We confirmed that under TG treatment, a reduction of the SLC38A2 transmembrane pool (co-stained with phalloidin, F-actin) was seen as previously demonstrated (Fig. S2D).^24^

To identify which intracellular vesicles contained SLC38A2, we co-stained MCF7 with an early endosome marker protein (RAB5) and the proteasome marker LMP2, where SLC38A2 is degraded.^25^ We found that SLC38A2 partially colocalised with these markers in normal conditions (Fig. S2E, F). However, when the cells were exposed to TG, the co-localisation between SLC38A2 and both RAB5 and LMP2 substantially decreased (Fig. S2E, F). These data suggest that, despite the mRNA induction of *SLC38A2* (Fig. 2d), diverse cell lines showed heterogeneous SLC38A2 response at protein levels in response to different stresses (including TG) and a shift of SLC38A2 protein from the TGN and plasma membrane to another intracellular compartment during induction and degradation (Fig. S2G).

### SLC38A2 is degraded by autophagy via LAMP1 rather than LAMP2

SLC38A2 has been shown to undergo degradation through the proteasome system.^25^ However, it is known that long-lived transmembrane proteins more frequently undergo degradation through autophagy.^26^ Recent work in Hela cells has shown that SLC38A2’s adaptive response to nutrient starvation requires retromer-mediated endosomal sorting, which prevents SLC38A2 being sequestered in LAMP1-positive lysosomes.^27^

**Fig. 3:**
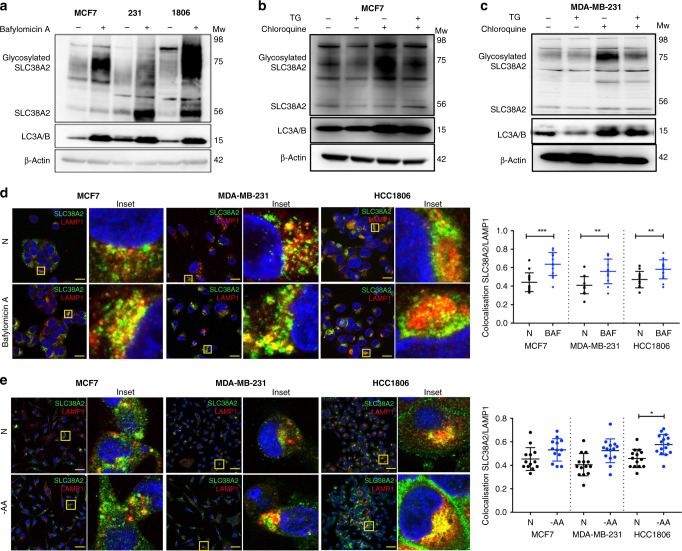
SLC38A2 is degraded by autophagy and co-localises with LAMP1. **a** Representative Western blots of SLC38A2 in three different breast cancer cell lines with or without Bafilomycin A1 treatment. β-actin is shown as a loading control. *N* = 3 **b**, **c** MCF7 cells (**b**) and MDA-MB-231 (**c**) were treated with thapsigargin (TG, 1 μm) or chloroquine (30 mM) or both with for 8 h. β-actin is shown as a loading control. *N* = 3 **d** Representative confocal images of SLC38A2 (green) and LAMP1 (red) in fixed MCF7 (left), MDA-MB-231 (middle) and HCC1806 (right) after Bafilomycin A1 treatment (Scale bars 15 μm). Pearson’s test co-localisation analysis of SLC38A2 and LAMP1 in three different breast cancer cell lines during Bafilomycin A1 treatment. Scheme of the analysis procedure: Circular patches surrounding a cell or a group of cell or manual evaluation SLC38A2 levels (ROI) and non-SLC38A2 signal (random ROI) were selected. Co-localisation values were calculated using a pixel-wise Pearson’s test. Frequency quantification of Pearson’s test values (−1: opposing, 0: no and 1: maximum co-localisation) for SLC38A2 vs. LAMP1 in the three breast cancer cell lines. Each dot represents a different measure. Aligned dot plot, SD; unpaired Student *t*-test. **p < 0.01, ***p < 0.001. *N* = 3. **e** Representative confocal images of SLC38A2 (green) and LAMP1 (red) in fixed MCF7 (left), MDA-MB-231 (middle) and HCC1806 (right) after AA starvation for 24 h (Scale bars 15 μm). On the right, Pearson’s test co-localisation analysis as mentioned above. Aligned dot plot, SD; unpaired Student *t*-test. *p < 0.05. *N* = 3.

Considering the heterogeneity of response to the stresses, here we assessed the route of SLC38A2 degradation by treating MCF7, MDA-MB-231 and HCC1806 with Bafilomycin A1, a pharmacological inhibitor of autophagosome-lysosome fusion.^28^ The levels of SLC38A2 increased consistently after Bafilomycin A1 treatment in all cell lines, suggesting lysosome-mediated degradation of SLC38A2 was also important in these breast cancer cell lines (Fig. 3a). The levels of SLC38A2 accumulation under Bafilomycin A1 reached higher levels compared to the induction seen under AA deprivation alone in HCC1806 (Fig. S3A). MCF7 and MDA-MB-231 cells were treated with TG and chloroquine, which is an inhibitor of autophagy.^29^ TG treatment caused a decrease of SLC38A2 as previously described, whereas treatment with chloroquine resulted in the accumulation of SLC38A2 and was able to rescue the TG-induced degradation of endogenous SLC38A2 (Fig. 3b, c).

Confocal imaging showed that SLC38A2 partially colocalises with LAMP1 under basal conditions (Fig. 3d), but this co-localisation was enhanced when the autophagic flux was blocked by Bafilomycin A1 (Fig. 3d, insets). Analysis of Biogrid immunoprecipitation repository confirmed the interaction between SLC38A2 and LAMP1, as well as other TGN proteins and RAB5^30^ (Fig. S3B). The same pattern of co-localisation upon Bafilomycin A1 treatment was not seen with LAMP2 (Fig. S3C), which has a predominant role in non-selective autophagy and in chaperone-mediated autophagy (for the LAMP2A isoform).^31^

As SLC38A2 has been previously reported to act as a transceptor,^32^ we hypothesised that SLC38A2 might serve as an AAT sensor in the lysosomes during AA starvation. However, despite an increase in SLC38A2 protein, no differences were seen in SLC38A2 and LAMP1 co-localisation during AA starvation compared to standard medium, apart from HCC1806 (Fig. 3e).

These data confirmed the co-localisation of SLC38A2 in the lysosomes, implicating them as a site of degradation for SLC38A2, but it is unclear whether SLC38A2 acts as an AA sensor in this compartment.

### SLC38A2 knockdown reduces glutamine consumption, mTORC1 signalling and regulates lysosomal biogenesis and autophagy in breast cancer cell lines

**Fig. 4:**
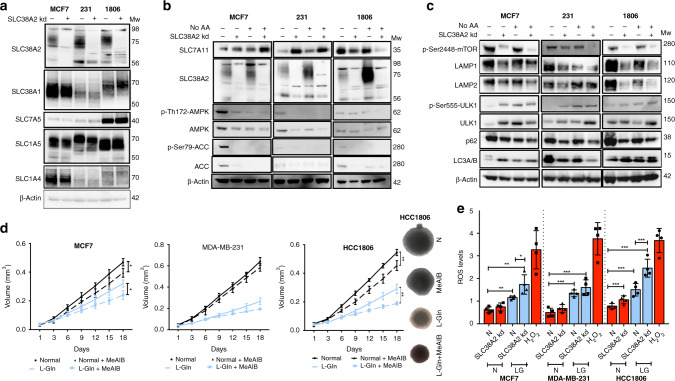
SLC38A2 knockdown modulates AMPK and mTORC1 pathway, sensitises MCF7 cells to low glutamine and increases ROS production. **a** Representative western blots of AATs levels after SLC38A2 knockdown in three different breast cancer cell lines. β-actin is shown as a loading control. *N* = 3. **b** Representative Western blots of three different breast cancer cell lines on the effects of siRNA SLC38A2 knockdown on the energy sensor AMPK and its downstream ACC and SLC7A11 in medium with or without aminoacid (AA). β-actin is shown as a loading control. *N* = 3 **b** Representative western blots of three different breast cancer cell lines on the effects of siRNA SLC38A2 knockdown on mTORC1 and autophagy pathway in medium with or without AA. β-actin is shown as a loading control. *N* = 3 **c** Graph of the effect of the System A (SLC38A1/2) inhibitor MeAIB (10 mM) treatment on MCF7, MDA-MB-231 and HCC1806 spheroid growth in normal (4 mM, black lines) or low glutamine medium (1 mM, blue lines). Error bars indicate SD. *P < 0.05, **P < 0.01, ***P < 0.001; two-way ANOVA. *N* = 3. On the right representative images of HCC1806 spheroids after the indicated treatments. **d** ROS levels quantification in three different breast cancer cell lines in normal (4 mM, N, red) or low glutamine medium (1 mM, LG, blue) with or without SLC38A2 knockdown. H_2_O_2_ treatment is shown as positive control. Error bars, SD. *p < 0.05, **p < 0.01. **p < 0.001, One-way ANOVA. *N* = 4.

Due to its localisation in the lysosomes and the role of AAT in modulating mTOR,^33^ we decided to investigate the effect of SLC38A2 in modulating autophagy and lysosomal function. We knocked down SLC38A2 with a siRNA pool in MCF7, MDA-MB-231 and HCC1806 cells. SLC38A2 knockdown did not cause a compensatory increase in other AATs, including SLC1A5 (Fig. 4a). However, SLC38A2 silencing caused a heterogeneous response of the cysteine transporter SLC7A11 levels, with an upregulation in MDA-MB-231 and MCF7, but a downregulation in HCC1806 (Fig. 4b). SLC38A2 depletion reduced the levels of lysosomal AMPK and p-AMPK as well as its downstream phospho-Acetyl-CoA carboxylase (p-ACC) and total ACC (Fig. 4b). The basal AMPK activation noted might be related to the drop-in glucose level in the experimental setting. The level of phosphorylated mTOR (p-mTOR) was also decreased in all the cell lines (Fig. 4c). This was concomitant with an increase in autophagy assessed by elevated ULK1 and p-ULK1 levels and decreased p62 in all cell lines (Fig. 4c). However, heterogeneity was seen after SLC38A2 knockdown in modulating the expression levels of LC3I/II with an increase in MCF7, a decrease in MDA-MB-231 and no effect in HCC1806 (Fig. 4c). These results confirmed the ability of SLC38A2, similar to other AATs, to modulate the mTORC1 signalling cascade as well as autophagy.^34^ Interestingly SLC38A2 knockdown decreases both the LAMP1 isoform mediating macroautophagy and the LAMP2 isoform mediating chaperone-mediated autophagy in all the breast cancer cell lines (Fig. 4c). These results suggest a role of SLC38A2 in modulating lysosomal biogenesis.

To investigate the role of SLC38A2 in breast cancer growth, we grew spheroids from three Gln- sensitive cell lines (MCF7, MDA-MB-231 and HCC1806) in normal medium and low Gln medium (Fig. 4d). Spheroids were then treated with system A (SLC38A1 and SLC38A2) inhibitor MeAIB. The pharmacological blockade of System A reduced spheroids growth in MCF7 and HCC1806 both in normal and in low-Gln medium (with a higher magnitude). The MDA-MB-231 spheroids were sensitive to Gln deprivation but not to System A inhibition, suggesting that System A is less relevant in this cell line, despite its sensitivity to Gln (Fig. 4d). SNAT2 depletion reduced colony formation, particularly under Gln deprivation in the three cell lines (Fig. S4A).

The effect of SLC38A2 knockdown or MeAIB was tested in 2D in the three different cell lines. The effect of the SNAT1/2 inhibitor showed similar pattern to SNAT2kd. The main effect for both treatments was mainly seen in low-Gln conditions except in HCC1806 where the SNAT2 levels are higher (Fig. S4B). We then investigated whether the reduced proliferation under SLC38A2 knockdown was linked to a reduced Gln intake. SLC38A2 knockdown decreased the Gln consumption in standard medium only in HCC1806 but not in MCF7 and MDA-MB-231. However, a clear effect was seen under lower Gln levels (1 mM) in HCC1806 and MCF7 cell lines, but not MDA-MB-231, after the SLC38A2 knockdown (Fig. S4C). This confirmed lack of a critical role of SLC38A2 in MDA-MB-231 cells under Gln deprivation.

### SLC38A2 knockdown increases ROS production in breast cancer cells during glutamine deprivation

It has been shown that TNBC cells can increase the uptake of Gln to indirectly support environmental cysteine acquisition via the SLC7A11/xCT antiporter.^4^ Glutamate, derived from Gln, and cysteine are substrates for the first and rate-limiting step in glutathione biosynthesis, a key intracellular anti-oxidant buffer against oxidative stress. We assessed whether SLC38A2 upregulation under Gln deprivation could maintain the Gln intake to modulate ROS production. Gln deprivation elevated ROS levels in all the three cell lines (Fig. 4e). The increase in ROS induced by low Gln levels was further elevated after reducing SLC38A2 protein levels by siRNA knockdown, particularly in MCF7 and HCC1806 but not in MDA-MB-231 where SLC38A2 knockdown was associated with an increased level of SLC7A11 (Fig. 4b).

Because the anti-oxidant *N*-acetylcysteine (NAC) was shown to mitigate ROS production in cancer cell lines,^35^ we postulated that exogenous supplementation of NAC might reduce the anti-growth effects of SLC38A2 blockade. We cultured MCF7, MDA-MB-231 and HCC1806 in normal and low Gln medium and we then knocked down SLC38A2 in combination with supplementation in the culture medium of 10 mM NAC. SLC38A2 knockdown reduced cell proliferation in normal medium only in HCC1806, where the SLC38A2 levels are higher, but the inhibitory effect in 2D culture was present in all the three cell lines in low Gln medium (Fig. S4D). Moreover, NAC supplementation partially rescued the inhibitory effect of SLC38A2 depletion in low Gln medium, particularly in HCC1806, where there was no compensation by SLC7A11 after SLC38A2 knockdown (Fig. 4b and S4D). Our results showed that the anti-tumour effect of SLC38A2 blockade in breast cancer cell lines, particularly in the HCC1806 TNBC cell line, in low Gln medium is partially mediated by oxidative stress.

### *SLC38A2* mRNA correlates with ATF4 and autophagy markers in the *Metabric* cohort

**Fig. 5:**
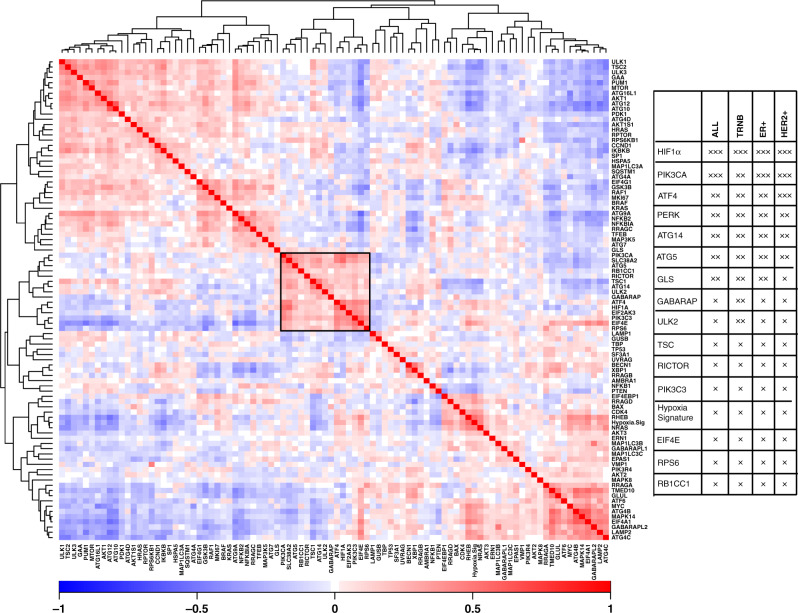
*SLC38A2* mRNA expression correlates with several genes of the mTORC1 pathway, autophagy and hypoxia in breast cancer patients. **a** Correlation heatmap of *SLC38A2* and other genes involved in the mTORC1 pathway, autophagy, as well as *HIF-1α*, and *ATF4*. *SLC38A2* co-expresses with *HIF-1α, PERK, ATF4* and *ATG5* (black box). Each square represents the Pearson correlation (r) between a pair of genes, calculated using microarray expression data from the Metabric cohort. Red colours indicate a high gene–gene correlation while the opposite is seen for the blue. **b** Table showing the significant correlation between *SLC38A2* expression and genes involved in the mTORC1 pathway, glutamine metabolism and autophagy in the breast cancer cohort.

As SLC38A2 was upregulated during different stresses and degraded by autophagy, we investigated if the expression of SLC38A2 correlated with ATF4 and autophagic markers in breast cancer patients. We analysed the gene expression data from 2433 breast cancer patients using the *Metabric* cohort,^36^ we found that *SLC38A2* mRNA abundance significantly correlated with the expression of *HIF-1α, ATF4*, and *PERK* and also other genes of the autophagy pathway such as *ATG5* (Fig. 5a, b) as well Gln metabolic genes such as glutaminase (*GLS*). Also, in the TNBC cohort of the *Metabric*, *SLC38A2* clustered with *HIF-1α, ATG5* and other autophagic genes like *GABARAP* and *ULK2* (Fig. S5A). Interestingly, *SLC38A2* co-expressed with other SNAT family members such as *SLC38A4/SNAT4*, *SLC38A5/SNAT5* and *SNAT7* (Fig. S5B) but not *SLC1A5* or *SLC7A5*. A significant, but weak, correlation was also seen between *SLC38A2* and *SLC7A11* mRNA expression (Fig. S5C).

### SLC38A2 protein is related to poor clinical outcome in breast cancer and particularly TNBC patients

**Fig. 6:**
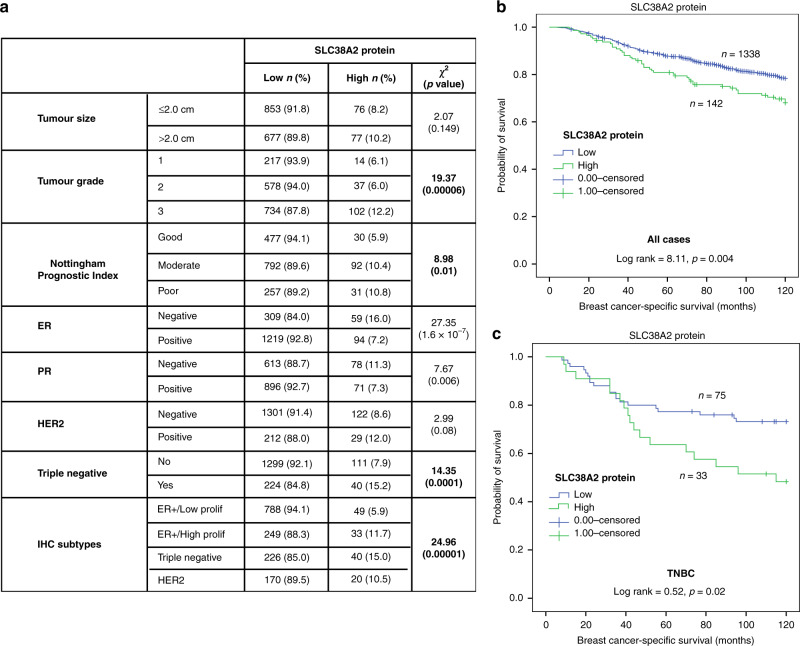
SLC38A2 expression and breast cancer patient outcome. **a** Correlation between SLC38A2 protein expression in breast cancer with different clinicopathological parameters in the breast cancer cohort. **b**, **c** Kaplan–Meier curves show that high expression of SLC38A2 protein in breast cancer tissues is associated with shorter breast cancer specific survival in the whole series (**b**), and in TNBC subgroup (**c**).

To examine the role of SLC38A2 in breast cancer patients, we stained a TMA of 1,685 primary breast cancer samples. SLC38A2 protein expression was observed, predominantly in the cytoplasm of invasive breast cancer cells, with expression levels varying from absent to high (Fig. S6A). High SLC38A2 expression (>105 H-score) was observed in 153/1685 (9%) of cases, while low expression (≤105 H-score) was observed in 1532/1685 (91%) of cases. High SLC38A2 expression was related to younger patient age, high tumour grade, moderate/poor Nottingham Prognostic Index and TNBC (all *p* < 0.01), but not with lymph node status (Fig. 6a and Fig. S6B).

Regarding breast cancer metastatic sites, high SLC38A2 protein levels were associated with the development of distant metastases in the brain (*p* = 0.005) but not at other sites (Fig. S6C). We then assessed the correlation of SLC38A2 protein with three previously analysed solute carrier (SLC) clusters.^37^ SLC38A2 protein levels correlated with the poor prognostic “high SLCs” (SLC1A5+/SLC7A5+/SLC3A2+) but not with the “low SLC” (SLC1A5−/SLC7A5−/SLC3A2−) nor the “high SLC1A5” cluster (SLC1A5+/SLC7A5−/SLC3A2−) (Fig. S6D).

Moreover, SLC38A2 expression was associated with poor BCSS in the entire cohort of breast cancer patients (Fig. 6b) and particularly in TNBC (*p* = 0.02) (Fig. 6c). There was no association between SLC38A2 protein and outcome in ER+ low, high proliferation or in HER2+ tumours (data not shown).

## Discussion

The role of Gln transporters on proliferation has not yet been studied extensively in TNBC.^15^ In this study, we showed that the system A transporters (SNAT1 and SLC38A2) are highly expressed in breast cancer cell lines. Interestingly, the cell lines showing high levels of AATs, including SLC38A2, are more sensitive to Gln deprivation and this dependency is also partially mediated by SLC38A2. We demonstrate that the SLC38A2 anti-tumour effect is partially mediated by oxidative stress and that high SLC38A2 protein expression in a large clinical cohort correlates with poor BCSS in patients and particularly those with TNBC.

Our results confirmed that Gln sensitivity is overall more prevalent in TNBC subtypes cell lines^38^ but also ER+ cell lines (MCF7) can show Gln sensitivity.^4,39^ These data suggest that the early metabolic responses of cancer cells to microenvironment stresses (such as nutrient deprivation) might be shared by different subtypes and going beyond the given genetic background of that cell line.

We found that cell lines with the highest induction of Gln transporters are also more sensitive to Gln deprivation and System A inhibition. This seems to be reflective of an upregulation of the whole Gln metabolic pathway, as these Gln-sensitive breast cancer cell lines are also more sensitive to drugs inhibiting this pathway such as aminotransferase inhibitors^39^ or glutaminase inhibitors.^6^

TNBC subtypes have been previously linked to increased Gln sensitivity due to the increased activity of the MYC pathway in this subtype compared to others.^40^ However, ER+ breast cancer cell lines with high levels of c-MYC are also dependent on Gln for their survival and growth^41^ and several c-Myc target AATs, including SLC1A5 and SLC7A5,^42,43^ correlate with poor survival in ER+ breast cancer, but not TNBC.^17,37^ Interestingly, we found that the SLC38A2 protein, which is a target for hypoxia-inducible factor-α (HIF1- α), but not c-Myc^12^ correlates with poor survival only in TNBC. A possible explanation could be that the activation of HIF gene networks is particularly robust in TNBC patients.^44^ Moreover, the XBP1 transcription factor, one of the main branches of the unfolded protein response (UPR) is specifically activated in TNBC tumours and transcriptionally cooperates with HIF-1α.^45^ Both the ATF4 upregulation under ER stress^46^ and HIF-1α and HIF-2α upregulation under hypoxia can promote the transcription of several AATs including SLC7A5 and SLC38A2.^12,47^ Moreover, HIF-1α and c-Myc can modulate adaptive responses to hypoxic environments.^48^ Thus, TNBC might be more dependent on Gln metabolism, due to the synergy between HIF-1α, XBP1 and c-Myc in modulating Gln metabolism, as well as AAT upregulation.

We showed that *SLC38A2* mRNA is highly abundant in breast cancer cell lines, as also seen in Hela and osteosarcoma cell lines.^10^ However, its abundance is tightly controlled by several mechanisms at the transcriptional^49^ and post-translational level.^50^ SLC38A2 protein shows marked heterogeneity in the levels of glycosylation; it undergoes proteasomal degradation under unsaturated fatty acid stress conditions^25^ and ubiquitination in response to ER-stress.^24^ The role of post-translational modifications in the SLC38A2 protein might account for the discrepancy between our previous results showing high *SLC38A2* mRNA is relevant in ER+ breast cancer tumours,^12^ while high SLC38A2 protein is relevant in TNBC. Similarly, this might be the cause of the difference in term of the lack of correlation between *SLC38A2 and SLC1A5 at* mRNA and the positive correlation seen at protein levels between the SLC38A2 and SLC1A5.

We also confirmed a route for SLC38A2 degradation via autophagy in breast cancer cell lines. The degradation of transporters, such as SLC38A2, through the activation of macroautophagy, might provide a molecular link between metabolic stress and cell death.^51^ However, the presence of SLC38A2 in lysosomes may just reflect its abundance and its turnover in the endocytosis pathway. Due to the similar localisation in LAMP1 vesicles, it would be interesting to investigate whether SLC38A2 might have a potential role also in the lysosomes by supplying the cytosolic AA derived from intracellular (autophagy) stores, as suggested for SLC38A7/SNAT7.^52^

Our results suggest that targeting SLC38A2 in Gln-sensitive cells reduces proliferation by impairing their ability to uptake sufficient Gln and potentially other neutral AA substrates of SLC38A2 from the extracellular environment. Also, the intracellular Gln concentration will drive the exchange of other AAs, e.g. leucine, isoleucine. However, a lack of anti-tumour effect in spheroids was seen in the Gln-sensitive cell MDA-MB-231 cell line in response to SLC38A2 inhibition. This could be potentially related to the higher utilisation of extracellular (macropinocytosis/necrocytosis) or intracellular AA pool from this cell line. The KRAS-mutant MDA-MB-231 cells are robustly macropinocytic amongst a wide variety of breast cancer cells^53^ and the autophagy pathway is still functional under glutamine starvation and SLC38A2 knockdown (Fig. 4c).

We have previously shown that SLC38A2 knockdown can modulate mTORC1 and reduce mitochondrial respiration, particularly in low Gln medium.^12^ In the present study, targeting SLC38A2 also reduces the lysosome energy sensor AMPK and p-AMPK, probably due to the reduced AA pool,^54^ and increased ROS production in low Gln medium. The increase in ROS was likely due to impaired Gln metabolism and cysteine/glutamate exchange, as has previously been seen for SLC1A5.^35,55^ However, in contrast to SLC1A5, we found an increase in ROS production mainly in low Gln medium after SLC38A2 knockdown, confirming recent suggestions that SLC38A2 acts as a rescue transporter providing AA intake under stress conditions.^9^

The role of SLC38A2 in maintaining mitochondrial function and ROS production in low Gln might be beyond the exchange with cysteine. In pancreatic cancer, SLC38A2 represents the major AAT for alanine, which is then deaminated to pyruvate (which can maintain the tricarboxylic acid cycle) in the presence of pyruvate carboxylase.^56^

Finally, our analysis of clinical data from a large breast cancer cohort suggests that high baseline SLC38A2 protein expression correlates with poor BCSS survival in patients with breast cancer and with TNBC. Moreover, tumours with high SLC38A2 expression were also commonly represented in the poor prognostic high SLC cluster (SLC1A5+/SLC7A5+/SLC3A2+). Interestingly, no significant association between the high SLC clusters and patient outcome was previously reported in TNBC.^37^ The TNBC subtype has still poorer outcome compared to other breast cancer subtypes due to aggressive clinical behaviour and a lack of recognised molecular targets for therapy.^57^ Blocking cellular Gln transport would potentially impart a greater impact on Gln metabolism in cancer cells than targeting downstream enzyme activity, given the extensive biological plasticity leveraged by cancer cells to maintain intracellular AA pools; new inhibitors with high affinity for AATs have been proposed.^58^ However, redundancy associated with other AATs after long-term silencing of SLC1A5 has been shown.^9^

Due to its transmembrane localisation and the vulnerability of the TNBC cell lines to Gln depletion and SLC38A2 knockdown, SLC38A2 is potentially a useful target for this cancer subtype. However, apart from the canonical competitive inhibitor of system A transport, MeAIB, no pharmacological agent specifically targeting SLC38A2 has been identified so far. Alternatively, SLC38A2 imaging for clinical selection by AA-based positron emission tomography radiotracers is also a promising approach^59^ that might inform treatment strategy. In conclusion, this study provides evidence for future investigation of SLC38A2 as a potential target for TNBC breast cancer patients and as a marker for sensitivity to Gln deprivation.

## Supplementary information

Supplemental Material

## Data Availability

The data presented in the current study are available upon reasonable request from the corresponding author.
