# Supplementary material for: Increased expression of glutamine transporter SNAT2/SLC38A2 promotes glutamine dependence and oxidative stress resistance, and is associated with worse prognosis in triple-negative breast cancer
Source: Br J Cancer. 2020 Oct 8;124(2):494–505. doi: 10.1038/s41416-020-01113-y (PMC7852531; doi:10.1038/s41416-020-01113-y)
Supplement: Supplementary file 1 — Supplemental Material [file 41416_2020_1113_MOESM1_ESM.pdf]

Supplementary Material

Supplementary Figure 1. ***SLC38A2* mRNA is highly expressed in breast cancer cell lines and is induced by glutamine starvation. A)** Analysis of several AATs from the CCLE in seven different breast cancer cell lines showing that *SLC38A2* mRNA is the more abundant transcript in these cell lines. The arrow indicates the direction from the AATs studied having higher mRNA expression (SLC38A2) to the ones with lower expression (2nd row, *SLC6A15*) **B)** The relative expression of different *AAT* genes in MCF7, MDA-MB-468, MDA-MB-231 and HCC1806 was analysed after culture in normal (N, 4 mM) and in low glutamine medium (LG, 1 mM) for 24 hours. Results were obtained by using the mean of the Ct values of each *AAT* transcripts after normalisation to housekeeping genes *(β-actin* and *RPL11*). Error bars, SD; unpaired Student t-test. * $p < 0.05$, ** $p < 0.01$; ***$p < 0.01$; $n = 3$. **C)** Breast cancer cell lines were grown in media with complete (N) or in 1 mM Glutamine (Gln) or 0.5 mM Gln in 10% dialysed FBS. The number of live cells per well was counted at day 3 and 6. Error bars indicate SD. *$P < 0.05$, **$P < 0.01$, ***$P < 0.001$; Unpaired t-test. N = 3.

Supplementary Figure 2. SLC38A2 is a trans-Golgi protein. A) Representative dual-colour STED image of fixed human MCF7 breast cancer cell line stained with the SLC38A2 antibody (green) and the trans-golgi marker TGN46 (red). Overview (main panel) and zoom-in (right) of area marked in overview. Insets: Examples of endosomal-like SLC38A2 pattern surrounded by TGN46, i.e. the Golgi membrane. Scale bars 5 µm (overview). **B)** Representative single-colour confocal image of fixed human MCF7 breast cancer cell line stained with the SLC38A2 antibody (green) and DAPI. Cells were first reverse transfected with siRNA against *SLC38A2* (siSLC38A2) or with scrambled controls and fixed after 5 days. Scale bars 10 µm. **C)** Representative confocal images of MDA-MB-231. Cells were fixed and stained with SLC38A2 (green) and with TGN46 (red) in normoxia (left column) and after 24 hours of: amino acid deprivation (EBSS medium, no AA;

second column), PP242 treatment (20 μm; third column) and thapsigargin treatment (8 h, fourth column, Scale bars 5 μm). Pearson's test colocalisation analysis of SLC38A2 at TGN46 during treatments. **D)** Representative confocal images of fixed MCF7 stained with the SLC38A2 antibody (green) and Phalloidin (F-actin, red) with or without thapsigargin (TG, 1 μm) treatment. Scale bars 10 μm. Pearson's test colocalisation analysis of SLC38A2 and Phalloidin with or without TG treatment. **E)** Representative confocal images of fixed MCF7 breast cancer cell line stained with the SLC38A2 antibody (green) and RAB5 (red) with (upper quadrant) or without TG (lower quadrant, 1 μm) treatment. Right quadrants represent insets. Scale bars 10 μm. Pearson's test colocalisation analysis of SLC38A2 and RAB5 with or without TG treatment **F)** Representative confocal images of fixed MCF7 cell line stained with the SLC38A2 antibody (green) and LMP2 (red) with (upper quadrant) or without thapsigargin (TG, lower quadrant, 1 μm) treatment. Right quadrants represent insets. Scale bars 10 μm. Pearson's test colocalisation analysis of SLC38A2 and LMP2 with or without TG treatment **G)** Proposed mechanism of SLC38A2 degradation under ER stress (TG treatment) in breast cancer cell lines

Supplementary Figure 3. SLC38A2 colocalises with LAMP1 but not LAMP2

A) HCC1806 cells were treated with AA starvation (24h, 10% dialysed FBS), Bafilomycin A1 for 24 hours. β-actin is shown as a loading control. N=3 **B)** Biogrid repository analysis of immunoprecipitation (IP) datasets showing that SLC38A2 interacts with several proteins including LAMP1. Other proteins located in the Golgi or with a role in cell trafficking were present as well. **C)** Representative confocal images of SLC38A2 and LAMP2 in MCF7 (left), MDA-MB-231 (middle) and HCC1806 (right) after Bafilomycin A1 treatment. Cells were fixed and stained with SLC38A2 (green) and with LAMP2 (red) (Scale bars 15 μm). Right quadrants represent insets.

Supplementary Figure 4. SLC38A2 knockdown decreases glutamine consumption and suppresses cancer growth partly via ROS production A) Clonogenic survival for MCF-7, MDA-

MB-231 and HCC1806 after SNAT2 siRNA knockdown with low glutamine (1 mM, LG, blue) or with normal glutamine (4 mM, N, red) levels. N=3. On the right representative images of HCC1806 colonies after the indicated treatments **B)** 2D growth curve of MCF-7, MDA-MB-231 and HCC1806 after SLC38A2 siRNA knockdown or MeAIB (10mM) treatment with low glutamine (1 mM, LG, blue) or with normal glutamine (4 mM, N, red) levels. A total of 10^5 cells were seeded. N=3 **(C)** Glutamine consumption was calculated in three different breast cancer cell lines cultured in normal glutamine (4 mM, N, red) or low glutamine medium (1 mM, LG, blue) with or without *SLC38A2* siRNA treatment. N=3 **(D)** 2D Growth curve of three breast cancer cell lines cultured in in normal (4 mM, N, red) or low glutamine (1 mM, LG, blue) medium with or without SLC38A2 knockdown and with or without N-acetylcysteine (10 mM, orange or violet) supplementation. A total of 10^5 cells were seeded. (n = 4), Scr, scrambled control. Error bars, SD; one-way ANOVA. * $p < 0.05$, ** $p < 0.01$, *** $p < 0.001$ for all the experiments.

Supplementary Figure 5. List of genes showing correlation with *SLC38A2* mRNA abundance in the Metabric cohort A) Correlation heatmap of *SLC38A2* and genes involved in autophagy and glutamine metabolism in TNBC. **B)** Correlation heatmap of *SLC38A2* and other AA transporters in the whole breast cancer cohort. Each square represents the Pearson correlation (r) between a pair of genes, calculated using microarray expression data from the Metabric cohort. Red colours indicate a high gene–gene correlation while the opposite is seen for the blue. **C)** Correlation plot of *SLC38A2* and *SLC7A11* transporter in the whole breast cancer cohort. Each dot represents a sample.

Supplementary Figure 6. SLC38A2 and breast cancer patient outcome A) Representative SLC38A2 IHC images of primary breast tumours to demonstrate strong, weak and absent staining. **B-C)** Clinicopathological associations of SLC38A2 expression in breast cancer in the Nottingham breast cancer cohort. **D)** Associations of SLC38A2 expression with the SLC clusters in breast cancer.

Suppl Fig 1

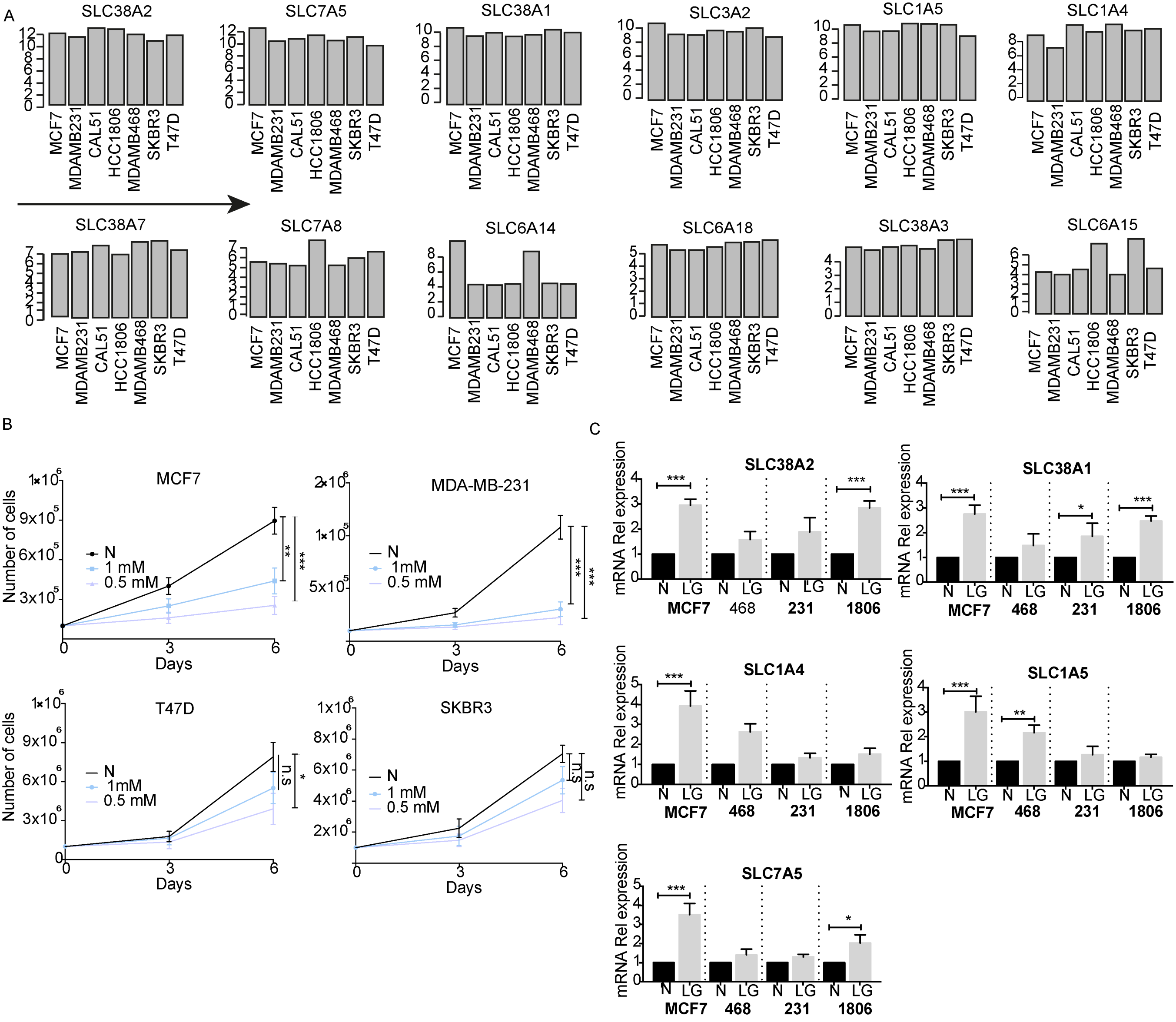
A
SLC38A2
SLC7A5
SLC38A1
SLC3A2
SLC1A5
SLC1A4
SLC38A7
SLC7A8
SLC6A14
SLC6A18
SLC38A3
SLC6A15
MCF7
MDAMB231
CAL51
HCC1806
MDAMB468
SKBR3
T47D
B
MCF7
MDA-MB-231
T47D
SKBR3
Number of cells
Days
N
1 mM
0.5 mM
n.s
C
SLC38A2
SLC38A1
SLC1A4
SLC1A5
SLC7A5
mRNA Rel expression
N
LG
MCF7
468
231
1806

Suppl Fig 2

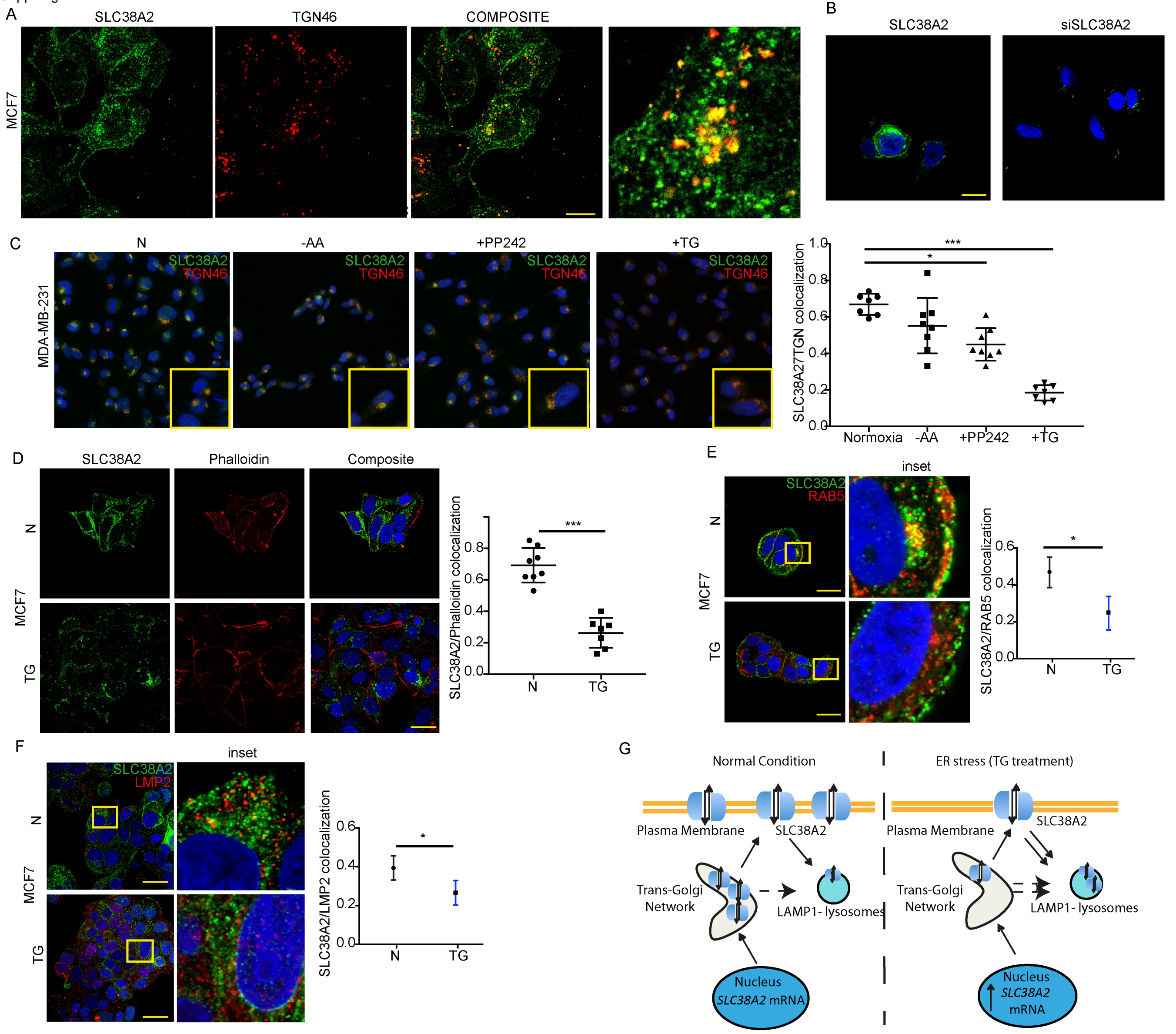

A
SLC38A2
TGN46
COMPOSITE
MCF7
B
SLC38A2
siSLC38A2
C
N
-AA
+PP242
+TG
SLC38A2
TGN46
MDA-MB-231
SLC38A2/TGN colocalization
1.0
0.8
0.6
0.4
0.2
0.0
***
*
Normoxia
-AA
+PP242
+TG
D
SLC38A2
Phalloidin
Composite
N
MCF7
TG
SLC38A2/Phalloidin colocalization
***
E
inset
SLC38A2
RAB5
SLC38A2/RAB5 colocalization
*
F
SLC38A2
LMP2
SLC38A2/LMP2 colocalization
G
Normal Condition
ER stress (TG treatment)
Plasma Membrane
SLC38A2
Trans-Golgi Network
LAMP1- lysosomes
Nucleus
SLC38A2 mRNA

Suppl Fig 3

A

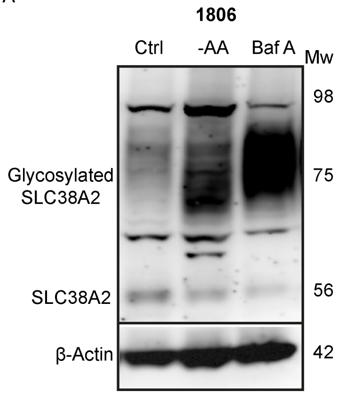

B

SLC38A2 - BIOGRID IP interaction dataset

**GOLT1B**	Golgi transport 1B
**TGOLN2**	Trans-Golgi network 2
**CHMP48**	Endosomal sorting
**RAB5**	Early-Endosomal sorting
**STX11**	Vescicular trafficking
**FLOT1**	Vescicular trafficking
**TUBA3A**	Neuronal trafficking
**LAMP1**	Lysosomal biogenesis

C

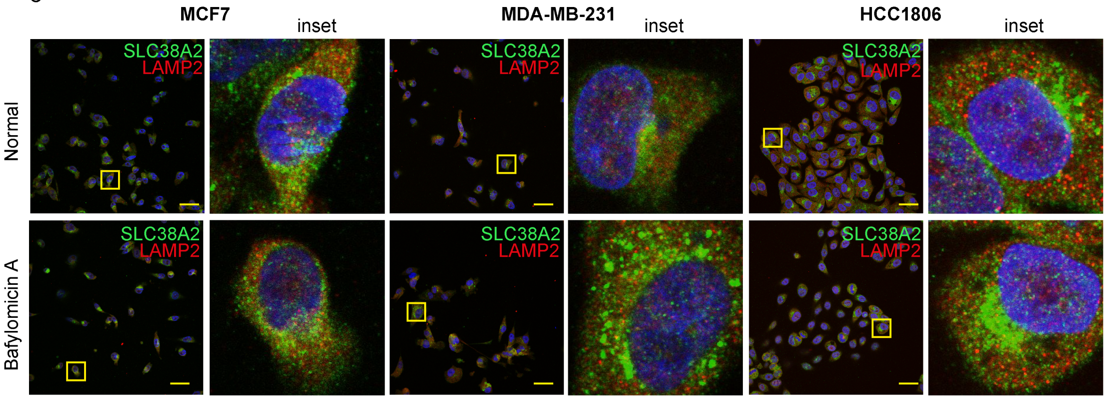

Suppl Fig 4

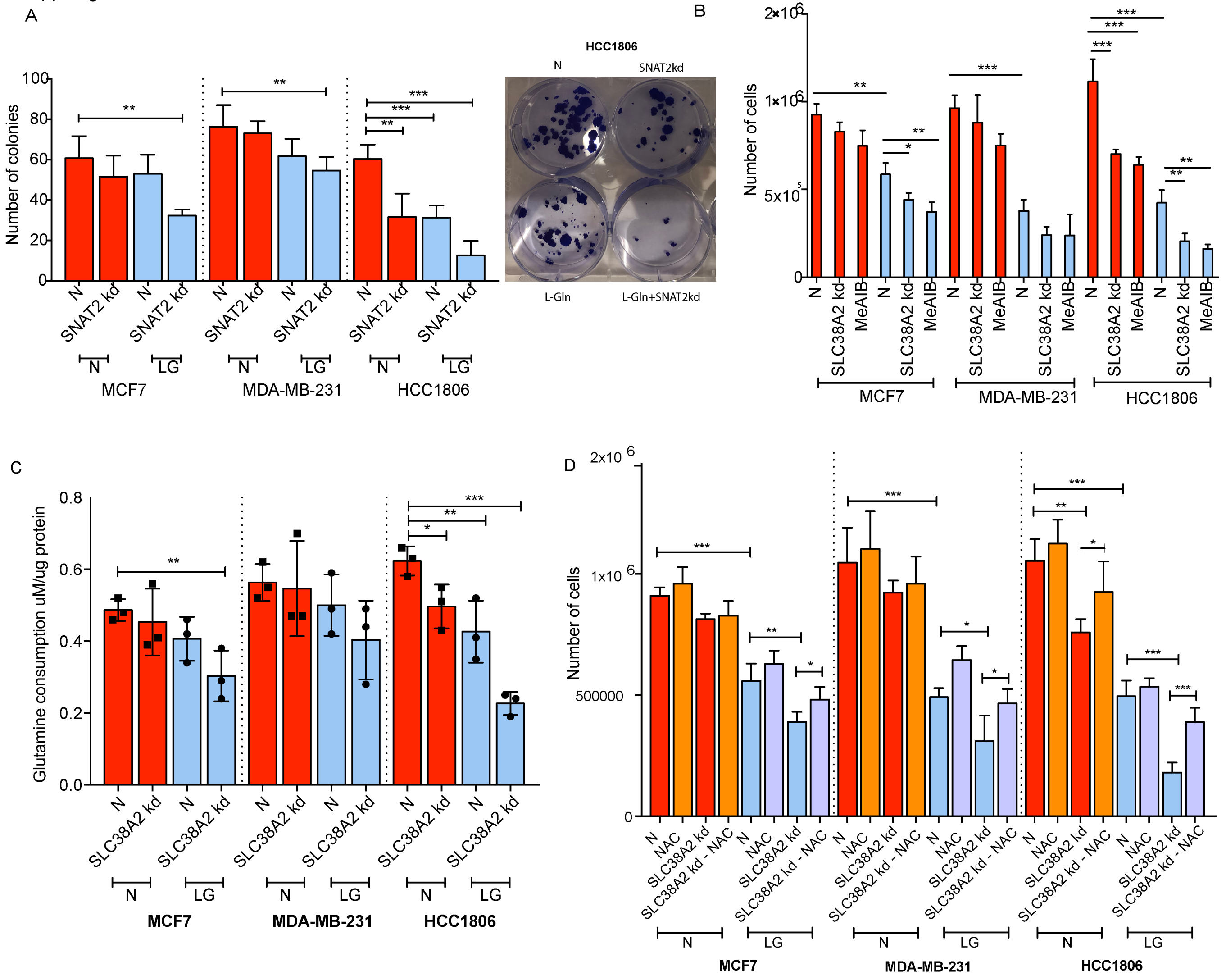

A
Number of colonies
MCF7
MDA-MB-231
HCC1806
N
SNAT2 kd
LG
HCC1806
SNAT2kd
L-Gln
L-Gln+SNAT2kd
B
Number of cells
SLC38A2 kd
MeAIB
C
Glutamine consumption uM/ug protein
D
NAC
SLC38A2 kd - NAC

Suppl Fig 5

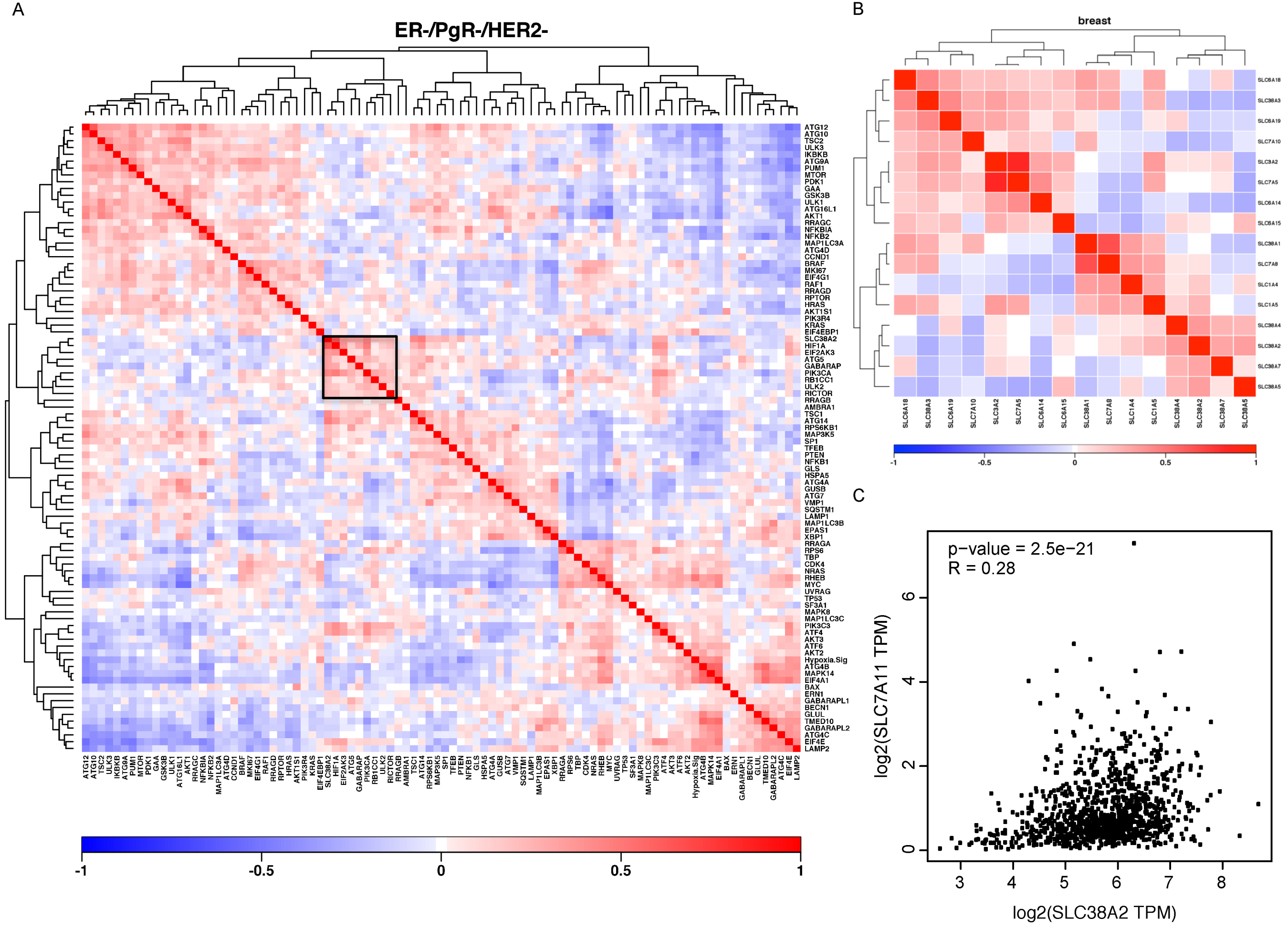

A
ER-/PgR-/HER2-
-1
-0.5
0
0.5
1
B
breast
SLC6A18
SLC38A3
SLC6A19
SLC7A10
SLC3A2
SLC7A5
SLC6A14
SLC6A15
SLC38A1
SLC7A8
SLC1A4
SLC1A5
SLC38A4
SLC38A2
SLC38A7
SLC38A5
C
p−value = 2.5e−21
R = 0.28
log2(SLC7A11 TPM)
log2(SLC38A2 TPM)

Suppl Fig 6

A

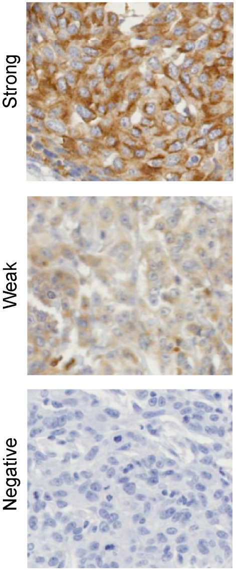

B

	SLC38A2 protein		
	Low n (%)	High n (%)	X2 (p-value)
Patient Age			
≤ 50	508 (88.8)	64 (11.2)	**4.63 (0.03)**
> 50	1023 (92.0)	89 (8.0)	
Lymph node status			
1	925 (90.9)	93 (9.1)	0.006 (0.99)
2	463 (91.0)	46 (9.0)	
3	138 (90.8)	14 (9.2)	
Histological type			
Ductal (including mixed)	1347 (90.8)	136 (9.2)	2.35 (0.79)
Lobular	99 (89.2)	12 (10.8)	
Medullary-like	25 (96.2)	1 (3.8)	
Miscellaneous	7 (100.0)	0 (0.0)	
Special type	50 (92.6)	4 (7.4)	
Tubular	2 (100.0)	0 (0.0)	
HER2+	170 (89.5)	20 (10.5)	

C

	SLC38A2 protein		
	Low n (%)	High n (%)	X2 (p-value)
**Site of distant metastasis**			
Bone			
No	436 (84.7)	79 (15.3)	0.037 (0.847)
Yes	122 (85.3)	21 (14.7)	
Liver			
No	481 (85.3)	83 (14.7)	0.709 (0.40)
Yes	77 (81.9)	17 (18.1)	
Brain			
No	531 (85.8)	88 (14.2)	**7.80 (0.005)**
Yes	27 (69.2)	12 (30.8)	
Lung			
No	506 (85.6)	85 (14.4)	2.993 (0.08)
Yes	52 (77.6)	15 (22.4)	

D

	SLC38A2 protein		
	Low n (%)	High n (%)	X2 (p-value)
**Low SLCs**	529 (94.8)	29 (5.2)	**39.21 (3.04×10^{-9})**
**High SLC1A5**	317 (92.2)	27 (7.8)	
**High SLCs**	165 (80.5)	40 (19.5)	
